# Supplementary material for: High resolution profiling of pathways of escape for SARS-CoV-2 spike-binding antibodies
Source: bioRxiv. 2020 Nov 16:2020.11.16.385278. Preprint. [Version 1] doi: 10.1101/2020.11.16.385278 (PMC7685320; doi:10.1101/2020.11.16.385278)
Supplement: 1 [file NIHPP2020.11.16.385278-supplement-1.pdf]

bioRxiv preprint doi: https://doi.org/10.1101/2020.11.16.385278; this version posted November 16, 2020. The copyright holder for this preprint (which was not certified by peer review) is the author/funder, who has granted bioRxiv a license to display the preprint in perpetuity. It is made available under aCC-BY 4.0 International license.

**Participant ID**	**Day(s) post symptom onset (p.s.o.)**	**Age**	**Gender**
1	27, 72	47	Female
2	31, 60	43	Female
3	29, 71	65	Male
4	31, 67	29	Male
5	31, 63	48	Female
6*	33, 76	64	Female
7	29, 74	22	Male
8	31, 67	31	Female
9	26, 55	56	Male
10	34, 67	28	Female
11	34, 62	30	Male
12	26, 58	36	Female
13	28, 66	65	Male
14	26, 64	65	Female
15	48, 77	52	Female
16	35, 69	52	Male
17	30, 57	36	Male
18	43, 73	29	Male

Table S1. Description of the COVID-19 patient samples used in this study. All patients exhibited mild symptoms not requiring hospitalization except for patient 6 (indicated by an asterisk), who had moderate symptoms requiring non-invasive ventilation or a high flow O_2 device.

bioRxiv preprint doi: https://doi.org/10.1101/2020.11.16.385278; this version posted November 16, 2020. The copyright holder for this preprint (which was not certified by peer review) is the author/funder, who has granted bioRxiv a license to display the preprint in perpetuity. It is made available under aCC-BY 4.0 International license.

A.

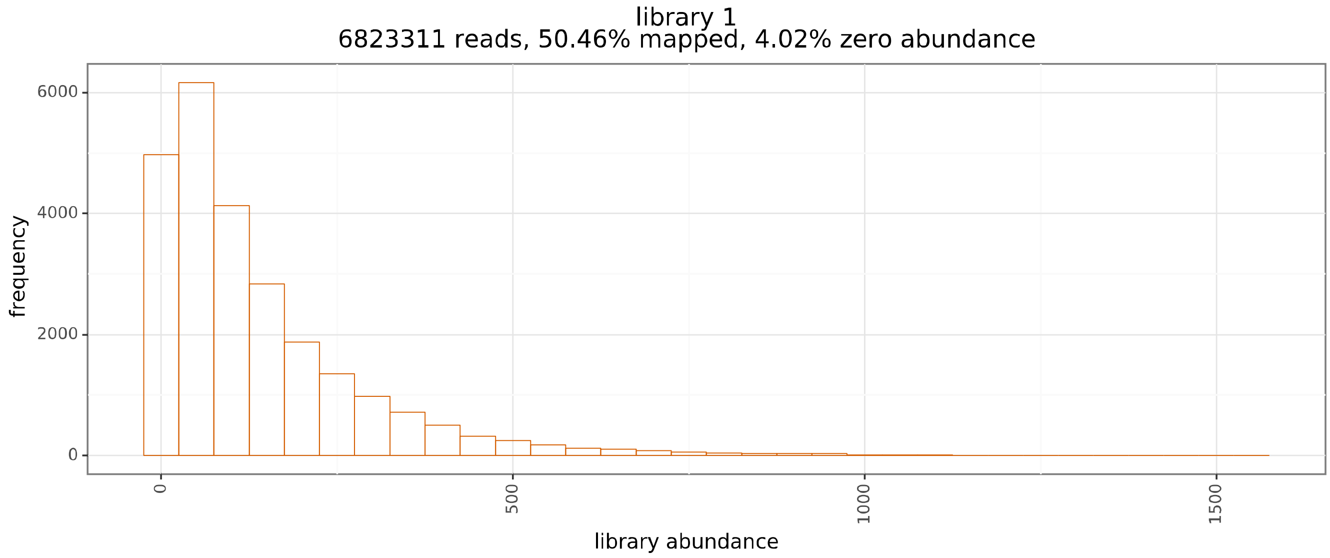

B.

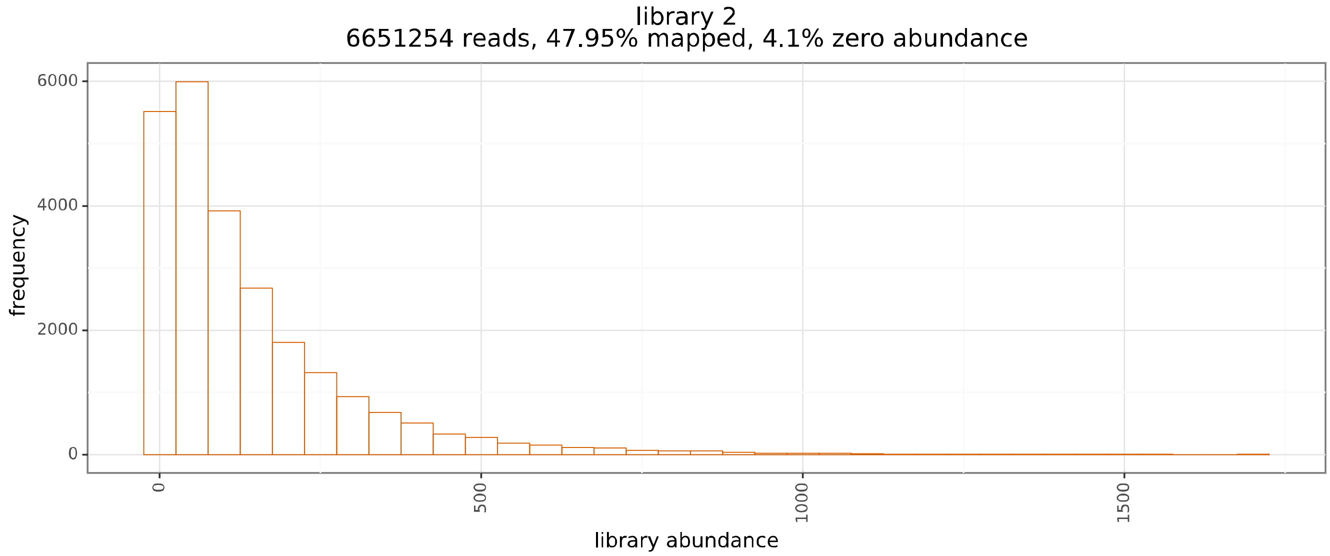

Figure S1. Distribution of sequenced peptides within biological replicate Spike Phage-DMS libraries. (A and B) Histogram showing the distribution of all sequenced peptides from a representative deep sequencing experiment for Spike Phage-DMS Library 1 (A) and Library 2 (B). Reads were stringently aligned to the reference library, allowing for 0 mismatches, and the proportion of unmapped reads is shown at the top. Additionally, the proportion of all non-sequenced peptides for each library is shown at the top.

bioRxiv preprint doi: https://doi.org/10.1101/2020.11.16.385278; this version posted November 16, 2020. The copyright holder for this preprint (which was not certified by peer review) is the author/funder, who has granted bioRxiv a license to display the preprint in perpetuity. It is made available under aCC-BY 4.0 International license.

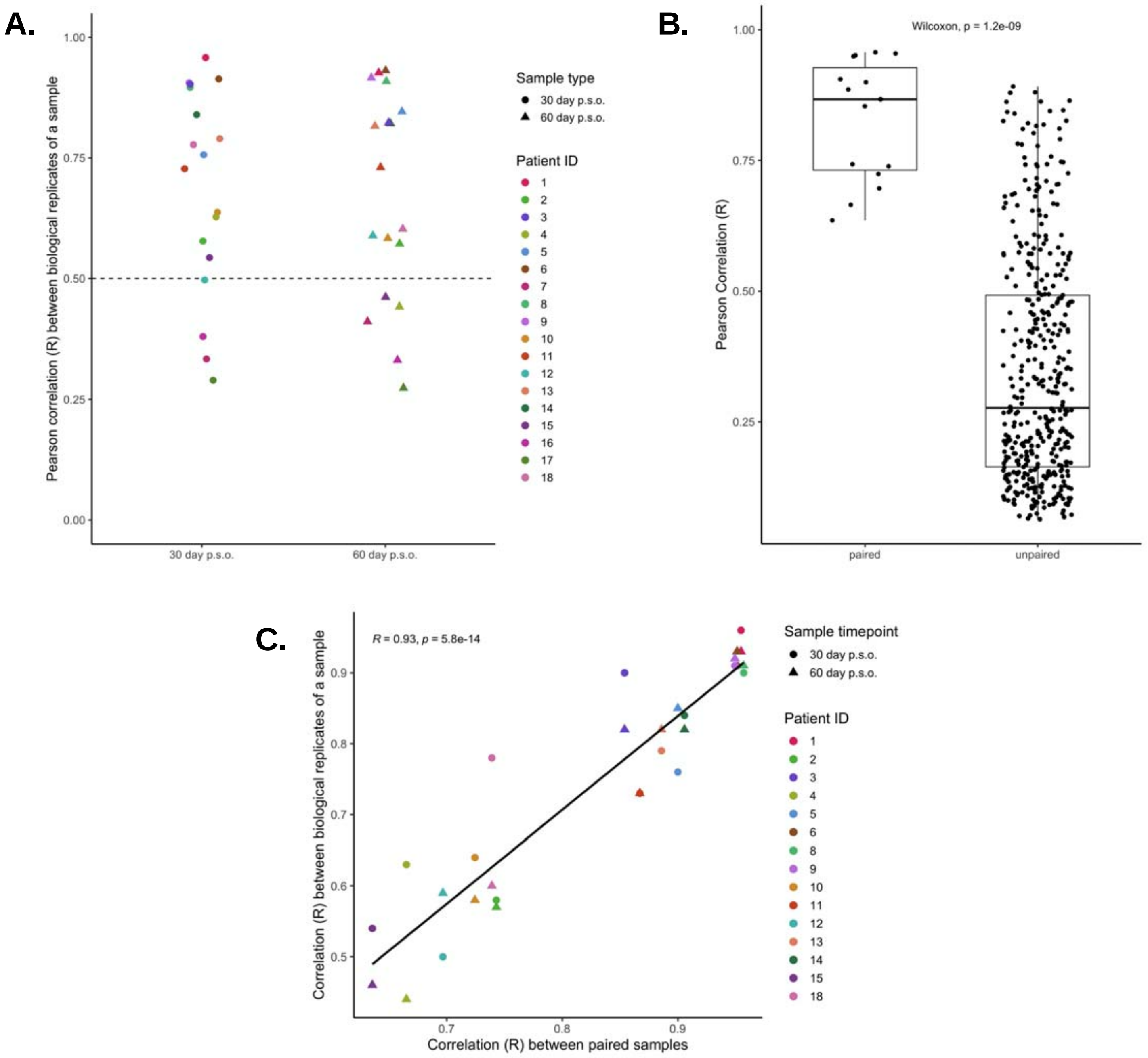

Figure S2. Reproducibility of peptide enrichment by plasma from COVID-19 patients. (A) Distribution of correlation values between peptide enrichment values for replicate experiments with samples from COVID-19 patients (Pearson's correlation coefficient, R). Each color corresponds to a unique patient or volunteer, and the shape of each dot represents the type of sample. A dotted line at y = 0.5 represents the cutoff used to determine whether samples were kept in the analysis. (B) Boxplots showing the distribution of correlation values between patient samples that were paired between the day 30 and 60 p.s.o. timepoints (on the left) or samples that were randomly paired and compared (on the right). (C) Relationship between the biological replicate correlation for a sample and its correlation with its paired timepoint. Each color corresponds to a unique patient, and the shape of each dot represents the type of sample. Pearson's correlation coefficient shown.

bioRxiv preprint doi: https://doi.org/10.1101/2020.11.16.385278; this version posted November 16, 2020. The copyright holder for this preprint (which was not certified by peer review) is the author/funder, who has granted bioRxiv a license to display the preprint in perpetuity. It is made available under aCC-BY 4.0 International license.

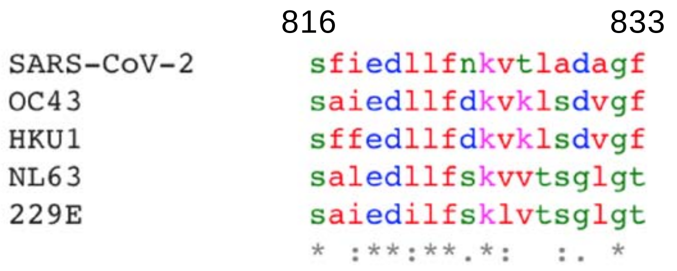

Figure S3. Multiple sequence alignment of the FP for SARS-CoV-2 and human endemic coronaviruses (OC43, HKU1, NL63, and 229E). Alignment was performed using Clustal Omega, and amino acids are colored according to physiochemical properties. GenBank accession numbers: YP_009724390.1, YP_009555241.1, YP_173238.1, YP_003767.1, and NP_073551.1, respectively.

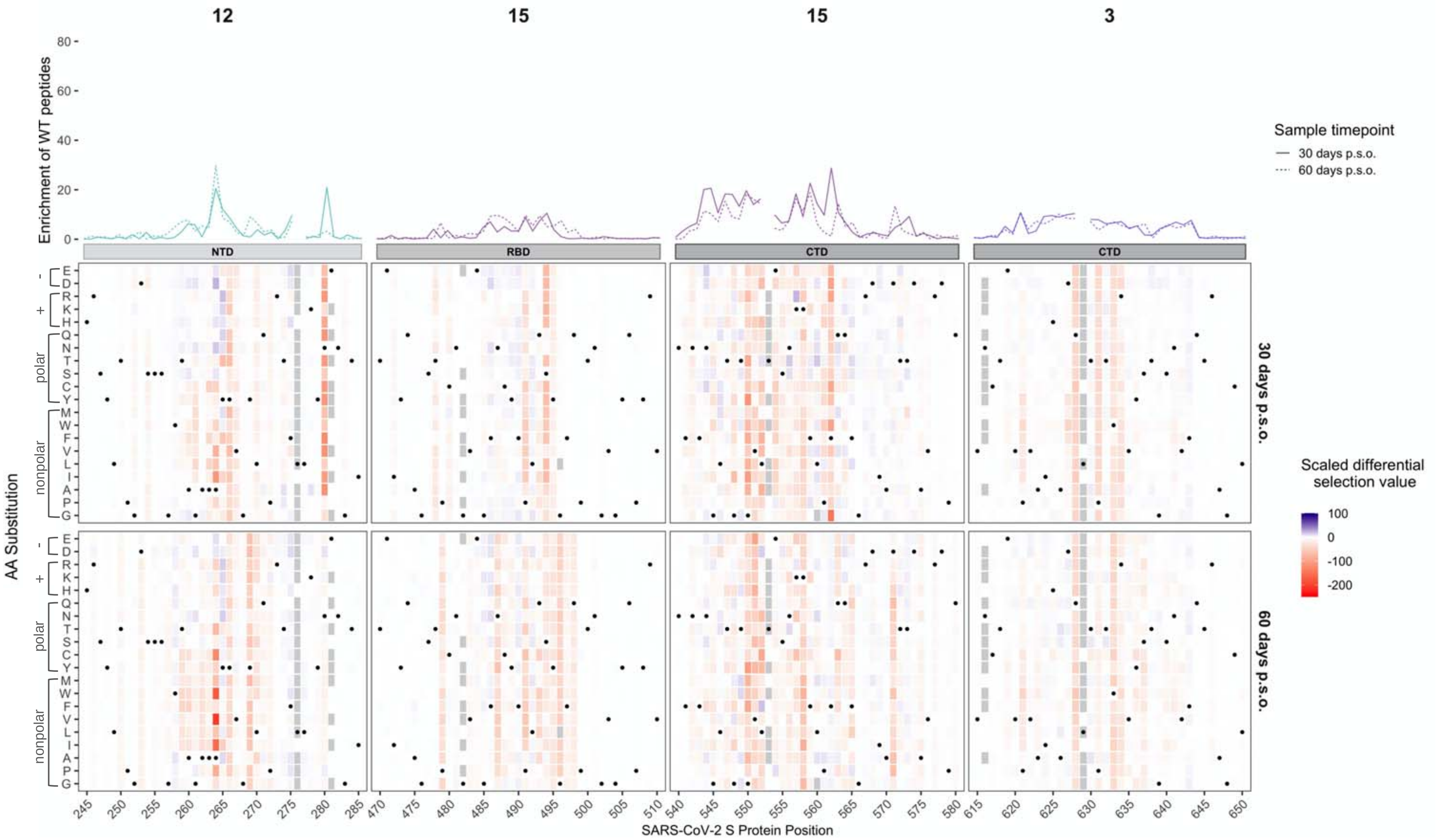

Figure S4. Effect of mutations on binding by COVID-19 patient plasma within various regions. Heatmaps depicting the effect of all mutations, as measured by scaled differential selection, at each site within the NTD, RBD, and CTD regions. Mutations enriched above the wildtype residue are colored blue and mutations depleted as compared to the wildtype residue are colored red. The wildtype residue is indicated with a black dot. Line plots showing the enrichment of wildtype peptides for each patient are shown above, with a solid line for patient samples taken at day 30 p.s.o. and a dashed line for patient samples taken at day 60 p.s.o. Peptides missing from the library are shown as grey boxes in the heatmaps and as breaks in the line plots.

bioRxiv preprint doi: https://doi.org/10.1101/2020.11.16.385278; this version posted November 16, 2020. The copyright holder for this preprint (which was not certified by peer review) is the author/funder, who has granted bioRxiv a license to display the preprint in perpetuity. It is made available under aCC-BY 4.0 International license.
